# Tumor Immune Microenvironment Heterogeneity and Key Immunoregulatory Gene Expression in Lung Cancer

**DOI:** 10.1155/ijog/8365959

**Published:** 2026-11-02

**Authors:** Jianghua Mao, Xue Zhang, Rongwei Yang, Xiaolin Wang

**Affiliations:** ^1^ Department of Radiology People′s Hospital of Chongqing Liangping District Chongqing China

**Keywords:** ISG15, lung cancer, single-cell RNA sequencing, TNFRSF18, tumor immune microenvironment

## Abstract

**Background:**

The aim of this study is to characterize the cellular heterogeneity and key immunoregulatory gene expression patterns of the tumor immune microenvironment (TIME) in lung cancer using single‐cell RNA sequencing (scRNA‐seq).

**Methods:**

The scRNA‐seq dataset GSM4453577 was retrieved from NCBI GEO. Unsupervised clustering, cell–cell communication analysis, pseudotime trajectory reconstruction, and GO/KEGG enrichment analyses were performed.

**Results:**

Seven immune cell types were annotated from 223 single cells after quality control. Macrophages demonstrated the strongest outgoing intercellular interaction intensity and showed relatively high SPP1‐associated expression‐based interaction scores, with macrophages and monocytes identified as the dominant SPP1 signal sources. ISG15 showed the broadest expression across all seven immune cell types (highest z‐scores in stem‐like/mast cells and neutrophils), whereas TNFRSF18 and TNFRSF4 exhibited similar descriptive expression trends during intermediate pseudotime stages (scaled 0–1), with TNFRSF18 enriched in stem‐like/mast cells (*z* = 1.93) and TNFRSF4 in mast cells (*z* = 2.22). T cell, NK cell, and Treg markers (CD3E, NKG7, and FOXP3) were below the detection threshold, consistent with the predominantly myeloid/B‐lineage immune infiltrate in this sample.

**Conclusion:**

ScRNA‐seq analysis of a single GEO sample (GSM4453577; 223 cells; PC1 explaining 5.3% of variance) suggested myeloid/B‐lineage immune cell heterogeneity in the lung cancer microenvironment and identified ISG15, TNFRSF18, and TNFRSF4 as candidates of potential immunoregulatory interest; external multisample validation is required before generalizing these findings.

## 1. Introduction

Lung cancer remains a leading cause of cancer incidence and mortality worldwide, and non–small cell lung cancer (NSCLC) accounts for approximately 85% of lung cancer cases [1]. Despite advances in diagnosis and treatment, NSCLC remains biologically heterogeneous. The tumor immune microenvironment (TIME), composed of immune cells and their interactions with other tumor‐associated cells, is an important component of this heterogeneity. Variation in immune cell abundance, functional states, and intercellular communication may influence local immune responses and the interpretation of potential therapeutic targets.

Conventional bulk transcriptomic analyses summarize gene expression across many cells and may obscure differences among immune cell populations. Single‐cell RNA sequencing (scRNA‐seq) enables immune subsets and candidate gene expression patterns to be examined at cellular resolution. Previous single‐cell studies of advanced NSCLC have demonstrated substantial diversity in tumor‐associated cell populations and microenvironmental states [2]. Studies have also mapped stromal heterogeneity in lung tumors [3], characterized conserved myeloid populations [4], profiled tumor‐infiltrating T‐cell states [5], and documented treatment‐associated tumor ecosystem changes [6]. Nevertheless, findings from individual samples, particularly those with low cell numbers, should be regarded as descriptive and exploratory rather than representative of all patients with NSCLC.

Among the immune populations of interest, macrophages and monocytes may contribute to expression‐based ligand–receptor (LR) interaction patterns. SPP1 has been studied in association with tumor‐associated macrophages (TAMs) in lung cancer [7]. Similarly, immunoregulatory molecules may exhibit different distributions across immune cell subsets. ISG15 is an interferon‐responsive gene with reported relevance to antitumor immune regulation in lung adenocarcinoma [8]. TNFRSF18 (glucocorticoid‐induced TNFR‐related protein [GITR]) and TNFRSF4 (OX40) are members of the tumor necrosis factor receptor superfamily and have been investigated as immunomodulatory receptors [9, 10]. Their biological roles, however, cannot be inferred solely from expression in a small observational dataset.

Additional candidate genes, including NOC2‐like nucleolar associated transcriptional repressor (NOC2L), hairy and enhancer of Split 4 (HES4), and stromal cell–derived Factor 4 (SDF4), may help describe transcriptional and cellular‐state differences in the observed immune populations. Computational tools can summarize cluster‐associated expression, estimate LR coexpression, and arrange cells along inferred pseudotime coordinates. Such outputs are useful for generating hypotheses but do not, without independent evidence, establish cellular identity, functional signaling, or a shared developmental trajectory across distinct immune lineages. This distinction is particularly important when some putative cell subsets contain only a few cells.

Here, we use the publicly available Gene Expression Omnibus (GEO) sample GSM4453577 to characterize immune cell heterogeneity and the expression patterns of selected immunoregulatory genes in a lung cancer specimen. We examine annotated immune cell groups, exploratory LR associations, functional enrichment, and computational pseudotime patterns. The objective is to identify descriptive features and candidate hypotheses for future investigation, without making patient‐level predictions or claiming independent biological validation.

## 2. Materials and Methods

### 2.1. ScRNA‐Seq Data Acquisition

ScRNA‐seq data were obtained from the NCBI GEO database under Accession Number GSM4453577, derived from lung cancer tumor tissue samples. The raw expression matrix contained 29,527 genes across 306 single cells.

### 2.2. Data Preprocessing and Quality Control

Data preprocessing was performed using Scanpy (Version 1.12) with Python 3.13. Quality control parameters included (1) filtering cells with mitochondrial gene percentage > 20%, (2) retaining cells with detected gene counts (nFeature_RNA) between 200 and 6000, and (3) filtering genes detected in fewer than three cells. After QC, 223 cells and 13,432 genes were retained for downstream analysis. The 83 excluded cells represent the combined outcome of these filters; criterion‐specific exclusion counts were not available from the documented summary. Pearson *r* = 0.95 in Figure 1A describes the association of nFeature_RNA with nCount_RNA and was not used as a doublet‐removal threshold. No dedicated doublet‐calling or ambient RNA correction procedure is documented; possible residual contamination and doublets are therefore limitations. Scanpy provides the computational framework used for single‐cell preprocessing and downstream analysis [11].

**Figure 1:**
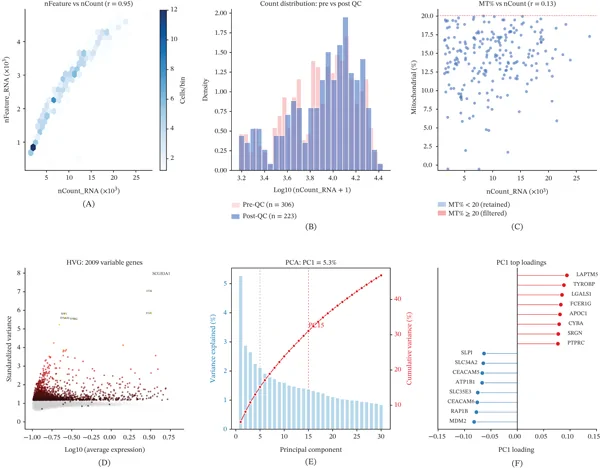
Quality control and dimensionality reduction of scRNA‐seq data from GSM4453577. (A) Hexbin density plot of nFeature_RNA versus nCount_RNA (Pearson *r* = 0.95), showing high cell density at low expression regions. (B) Frequency distribution histograms comparing nCount_RNA distribution before (*n* = 306, pink) and after (*n* = 223, blue) quality control filtering. (C) Quality control scatter plot of mitochondrial percentage versus nCount_RNA (Pearson *r* = 0.13). The red dashed line indicates the 20% MT threshold; blue points represent retained cells; red points represent filtered cells. (D) Highly variable gene selection plot showing standardized variance versus average expression. Red points indicate 2009 selected HVGs (Seurat V3); Top 6 HVGs are labeled. (E) Dual‐axis PCA elbow plot. Bars represent individual PC variance explained (left *y*‐axis, blue); red line represents cumulative variance (right *y*‐axis). PC1 explains 5.3% of variance; elbow at PC5; 15 PCs retained (cumulative 31.1%). (F) Diverging lollipop chart of PC1 loadings. Red: positive loadings (immune‐related genes); blue: negative loadings (epithelial/stromal genes). Top 8 genes from each direction are shown.

### 2.3. Normalization and Dimensionality Reduction

Count data were normalized to 10,000 total counts per cell followed by log1p transformation. Highly variable genes (HVGs) were identified using the Seurat V3 flavor method (*n*_top_genes = 2000) [12], supplemented with forced inclusion of key immune‐regulatory marker genes, yielding 2009 HVGs in total. Forced inclusion ensured that the following key immune‐regulatory marker genes were retained: CD79A, MS4A1, CD68, CSF1R, CD14, KIT, TPSAB1, CPA3, MS4A2, FCGR3A, S100A8, S100A9, SDC1, XBP1, ALDH1A1, SPP1, MIF, CD74, CXCR4, CD44, NOC2L, HES4, ISG15, TNFRSF18, TNFRSF4, and SDF4. Principal component analysis (PCA) was performed on scaled HVG expression data (max_value = 10). The Top 15 principal components were selected based on elbow plot analysis for downstream clustering and visualization. The selected immune markers were retained to facilitate descriptive annotation and candidate gene visualization, rather than to provide independent evidence of cluster identity. Fifteen PCs and a resolution of 2.0 were used as exploratory parameter choices; clustering robustness to alternative settings or exclusion of forced markers was not assessed.

### 2.4. Unsupervised Clustering and Cell Type Annotation

A *k*‐nearest neighbor graph was constructed using the first 15 PCs (*n*_neighbors = 15), followed by Leiden community detection at a resolution of 2.0, yielding 11 distinct clusters. Cell type annotation was performed by gene signature scoring using canonical immune cell markers: plasma cells (SDC1 and XBP1), monocytes (CD14, FCGR3A, S100A8, and S100A9), B cells (CD79A and MS4A1), macrophages (CD68, CSF1R, and CD14), neutrophils (S100A8), mast cells (KIT, TPSAB1, CPA3, and MS4A2), and stem‐like/mast cells (KIT and ALDH1A1). Each cell was assigned to the cell type with the highest module score. Two‐dimensional visualization was performed using uniform manifold approximation and projection (UMAP) and t‐distributed stochastic neighbor embedding (t‐SNE). These are provisional marker‐based annotations, particularly for the small KIT/ALDH1A1‐positive group termed “stem‐like/mast,” for which progenitor identity cannot be established from these markers alone. No independent reference‐based annotation was performed. Leiden is a graph‐based community detection approach used to define transcriptional clusters [13].

### 2.5. Cell–Cell Communication Analysis

LR pair communication analysis was conducted using a curated database of 16 immune‐relevant LR pairs, including SPP1–CD44, MIF–CD74, CSF1–CSF1R, CCL5–CCR1/3/5, CXCL12–CXCR4, TNF–TNFRSF1A/1B, IL1B–IL1R1/2, TGFB1–TGFBR1/2, VEGFA–FLT1/KDR, IL10–IL10RA/B, IFNG–IFNGR1/2, IL6–IL6R/IL6ST, CCL2–CCR2, LGALS9–HAVCR2/CD44, HMGB1–TLR4, and ANXA1–FPR1. For each LR pair, the expression‐based interaction score from sender cell type S to receiver cell type R was calculated as the product of average ligand expression in S and average receptor expression in R, using raw count data; these scores are uncalibrated and may be affected by sequencing depth. Total interaction counts and normalized communication intensities were computed across all sender–receiver pairs. They were compared descriptively across sender–receiver pairs without permutation‐based significance testing and should not be interpreted as experimentally validated signaling probabilities.

### 2.6. SPP1 Signaling Pathway Analysis

The SPP1 signaling network was specifically analyzed by mapping SPP1 ligand expression against its four known receptors: CD44, ITGAV, ITGB1, and ITGB3. Coexpression heatmaps were generated to visualize the SPP1‐receptor interaction landscape across the seven annotated immune cell types. Expression of key pathway genes (SPP1, MIF, CD74, CD44, CXCR4, ITGAV, ITGB1, and CD14) was examined across cell types, and Pearson correlation matrices were computed to describe pairwise expression associations; such associations alone cannot establish pathway coordination.

### 2.7. Functional Enrichment Analysis

Differentially expressed genes (DEGs) were identified using the Wilcoxon rank‐sum test across all 11 Leiden clusters (adjusted *p* < 0.05). A total of 182 unique DEGs were submitted to Enrichr for Gene Ontology (GO) enrichment analysis across three ontology categories: biological process (GO_Biological_Process_2023), cellular component (GO_Cellular_Component_2023), and molecular function (GO_Molecular_Function_2023). KEGG pathway enrichment analysis was performed using the KEGG_2021_Human database. Enrichment significance was assessed using the Benjamini–Hochberg adjusted *p* value, with −log10(FDR) used as the enrichment score. Genes were selected using adjusted *p* < 0.05; no additional absolute log2 fold‐change threshold was specified. The comparisons were cluster‐wise, and the enrichment background was the database default rather than a separately documented expressed gene universe. Accordingly, pathway rankings should be interpreted cautiously.

### 2.8. Pseudotime Trajectory Analysis

Diffusion map dimensionality reduction was applied to the HVG‐scaled expression matrix, followed by diffusion pseudotime (DPT) computation using the cell with the highest gene count as the root. Pseudotime values were scaled to a 0–1 range. Gene expression dynamics along pseudotime were analyzed using sliding window smoothing (window size = max(5, *n*_cells/25)) for six key genes: ISG15, NOC2L, HES4, TNFRSF18, TNFRSF4, and SDF4. Z‐score‐normalized expression heatmaps were generated across 40 pseudotime bins to visualize coordinated gene expression patterns. The root was selected computationally, not on the basis of lineage biology. Because distinct immune lineages were pooled, DPT here represents a descriptive embedding‐based ordering rather than a validated developmental trajectory; root‐choice sensitivity and formal temporal coexpression tests were not performed. DPT is a computational ordering method and does not, by itself, establish developmental ancestry [14].

### 2.9. Key Gene Expression Analysis

The expression patterns of six key immunoregulatory genes (ISG15, TNFRSF18, TNFRSF4, NOC2L, HES4, and SDF4) were characterized across all seven immune cell types. Spatial distribution on t‐SNE embeddings was visualized using feature plots with six distinct colormaps (Greens, Purples, OrRd, YlOrBr, Blues, and RdPu). Average expression per cell type was computed from the log‐normalized expression matrix, and z‐score normalization was applied for cross‐gene comparison.

## 3. Results

### 3.1. Quality Control and Dimensionality Reduction

ScRNA‐seq data from GSM4453577 were subjected to rigorous quality control (Figure 1). Hexbin density analysis (Figure 1A) confirmed a strong correlation between nFeature_RNA and nCount_RNA (Pearson *r* = 0.95). Frequency distribution comparison (Figure 1B) showed a reduction from 306 to 223 cells after filtering (MT% < 20%, nFeature 200–6000). The quality control scatter plot (Figure 1C) confirmed a weak correlation between mitochondrial percentage and total counts (*r* = 0.13), with cells exceeding the 20% MT threshold excluded. HVG selection (Figure 1D) identified 2009 HVGs using Seurat V3, with top variable genes prominently labeled. PCA elbow analysis (Figure 1E) demonstrated PC1 explaining 5.3% of variance with an inflection point at PC5; the first 15 PCs cumulatively explained 31.1% of variance. PC1 loading analysis (Figure 1F) revealed clear separation between immune‐related genes (positive loadings) and epithelial/stromal genes (negative loadings).

### 3.2. Immune Cell Clustering and Subpopulation Identification

Unsupervised Leiden clustering at a resolution of 2.0 with 15 PCs identified 11 distinct cell clusters (Figure 2). UMAP visualization with convex hulls (Figure 2A) delineated clear boundaries between the seven annotated cell types. t‐SNE analysis (Figure 2B) provided a complementary low‐dimensional representation with enhanced separation. Donut chart analysis (Figure 2C) revealed plasma cells as the dominant population (107 cells, 48.0%), followed by monocytes (44 cells, 19.7%), B cells (25 cells, 11.2%), macrophages (18 cells, 8.1%), neutrophils (13 cells, 5.8%), mast cells (8 cells, 3.6%), and stem‐like/mast cells (8 cells, 3.6%). Stacked bar analysis (Figure 2D) confirmed strong correspondence between Leiden clusters and annotated cell types. Marker gene dot plot (Figure 2E) validated cell type annotations through canonical marker expression patterns (CD79A, MS4A1 for B cells; CD68, CD14 for myeloid; KIT, TPSAB1 for mast cells; and SDC1, XBP1 for plasma cells). ISG15 violin plot (Figure 2F) demonstrated broad expression across all seven immune cell types, suggesting broad expression within this specimen. T‐cell markers (CD3E, CD4, and CD8A), NK cell markers (NKG7 and GNLY), and Treg markers (FOXP3) were below the detection threshold, consistent with the predominantly myeloid/B‐lineage immune infiltrate in this sample.

**Figure 2:**
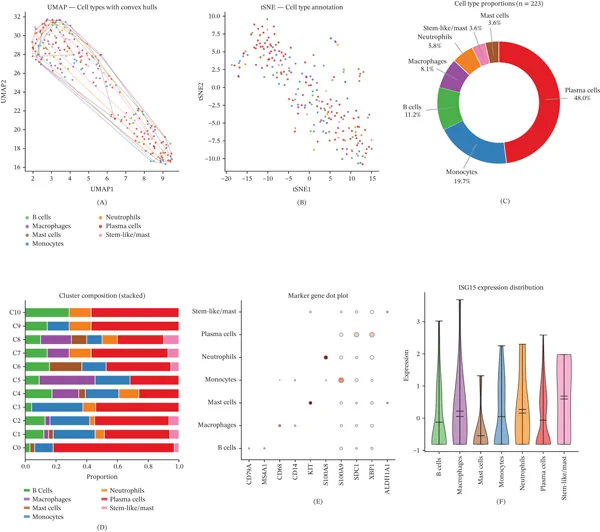
Single‐cell clustering and immune cell type annotation. (A) UMAP visualization of seven annotated immune cell types with convex hull outlines delineating cell‐type boundaries. (B) t‐SNE visualization of the same seven cell types, providing complementary low‐dimensional representation. (C) Donut chart showing cell‐type proportions among 223 QC‐passed cells. Plasma cells constitute the largest population (48.0%), followed by monocytes (19.7%) and B cells (11.2%). (D) Stacked horizontal bar chart of per‐cluster cell‐type composition, showing the correspondence between Leiden clusters and annotated cell types. (E) Dot plot of canonical immune marker gene expression (CD79A, MS4A1, CD68, CD14, KIT, S100A8, S100A9, SDC1, XBP1, and ALDH1A1). Dot size reflects the percentage of expressing cells; color intensity reflects mean expression level. (F) Violin plot of ISG15 expression distribution across the seven immune cell types.

### 3.3. Cell–Cell Communication Network Analysis

LR pair analysis using 16 curated immune LR pairs suggested putative LR coexpression patterns (Figure 3). The clustered interaction count heatmap (Figure 3A, YlOrRd colormap) identified plasma cells, monocytes, and macrophages as participating in the highest numbers of LR interactions, with Ward hierarchical clustering organizing sender–receiver patterns. Normalized communication intensity analysis (Figure 3B, coolwarm colormap) demonstrated that macrophages exhibited the highest estimated outgoing interaction score in this expression‐based model. Sender strength quantification (Figure 3C) ranked total outgoing signaling capacity across all seven cell types. Individual cell‐type communication profiles were visualized through three distinct chart types: bubble chart for the top sender (Figure 3D), vertical bar chart for the second‐ranked sender (Figure 3E), and connected dot plot with numeric annotations for the third‐ranked sender (Figure 3F), revealing cell‐type‐specific target preferences.

**Figure 3:**
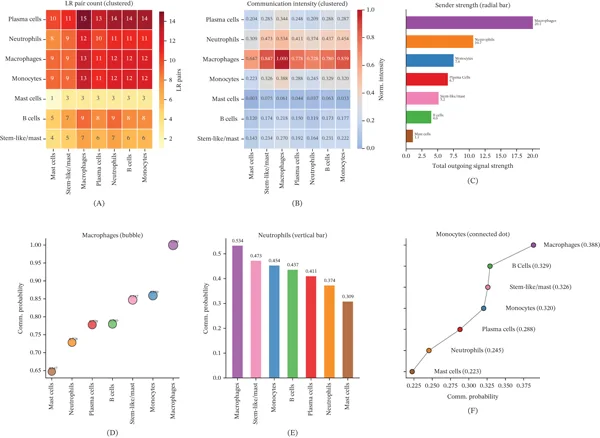
Cell–cell communication network analysis of immune cells. (A) Clustered heatmap of LR pair interaction counts between sender and receiver cell types, with row and column dendrograms from Ward hierarchical clustering. Color intensity represents the number of active LR pairs (YlOrRd colormap). (B) Clustered heatmap of normalized communication intensity (coolwarm colormap), showing expression‐based interaction score for each sender–receiver pair. (C) Radial bar chart of total outgoing signal strength by sender cell type, ranked by total communication output. (D) Bubble chart showing communication strength from the top sender to all receiver cell types, with bubble size proportional to expression‐based interaction score. (E) Vertical bar chart of outgoing expression‐based interaction score for the second‐ranked sender. (F) Connected dot plot showing expression‐based interaction score with numeric annotations for the third‐ranked sender.

### 3.4. SPP1 Signaling Pathway Analysis

The SPP1 signaling pathway was comprehensively characterized across immune cell populations (Figure 4). UMAP feature visualization (Figure 4A, OrRd colormap) showed SPP1 expression predominantly enriched in macrophages and monocytes, identifying these populations as primary SPP1 signal sources [7, 15]. SPP1 versus MIF coexpression scatter analysis (Figure 4B) showed a weak negative association (Pearson *r* = −0.08) between these two genes across cell types, with Pearson correlation coefficient and linear regression line. SPP1‐receptor coexpression heatmap analysis (Figure 4C, YlOrBr colormap) suggested macrophage‐associated coexpression scores through CD44 and integrin receptors (ITGAV, ITGB1, and ITGB3). CD74 raincloud plot (Figure 4D) showed the highest MIF receptor expression in neutrophils and plasma cells, compatible with potential MIF–CD74 receptor availability, without proving signaling activity in these populations. CD44 proportion analysis (Figure 4E) quantified the percentage of CD44‐positive cells in each immune cell type. Pearson correlation matrix analysis (Figure 4F) of eight SPP1 pathway genes (SPP1, MIF, CD74, CD44, CXCR4, ITGAV, ITGB1, and CD14) described pairwise associations, but did not establish a coordinated or functionally active SPP1–MIF–CD74–CD44 axis.

**Figure 4:**
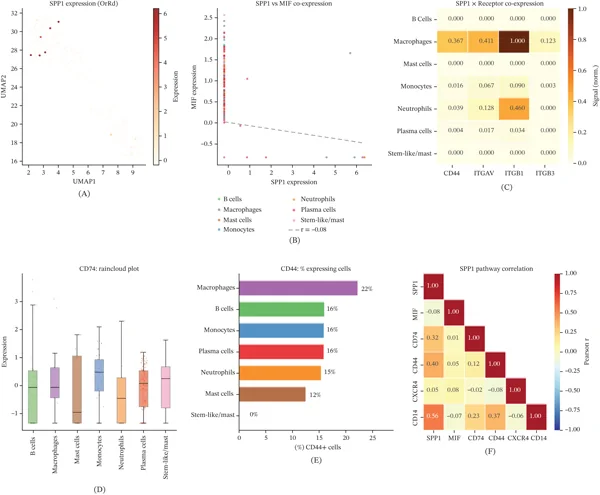
SPP1 signaling pathway and key immune gene expression analysis. (A) SPP1 expression feature plot on UMAP (OrRd colormap), showing enrichment in macrophage and monocyte populations. (B) SPP1 versus MIF coexpression scatter plot colored by cell type, with Pearson correlation coefficient and linear regression line, showing a weak negative expression association (*r* = −0.08), not evidence of pathway coordination. (C) SPP1‐receptor coexpression heatmap (YlOrBr colormap) showing normalized expression‐based interaction score between SPP1 ligand and each receptor (CD44, ITGAV, ITGB1, and ITGB3) across seven cell types. (D) Raincloud plot (violin + jittered points) of CD74 (MIF receptor) expression distribution across cell types. (E) Horizontal bar chart showing the percentage of CD44‐positive cells in each immune cell type. (F) Pearson correlation matrix of SPP1 pathway genes (SPP1, MIF, CD74, CD44, CXCR4, and CD14), showing upper‐triangle correlation values (RdYlBu_r colormap).

### 3.5. Functional Enrichment and Key Gene Expression Analysis

Differential expression analysis across 11 Leiden clusters identified 182 significant DEGs for functional enrichment (Figure 5). GO biological process lollipop chart (Figure 5A) identified top enriched terms including cytoplasmic translation, protein targeting to ER, and mRNA catabolic processes. GO cellular component gradient bar analysis (Figure 5B, Greens) revealed enrichment in cytosolic ribosome, ribosomal subunit, and spliceosomal complex categories. GO molecular function vertical bar analysis (Figure 5C) showed enrichment in structural constituent of ribosome and RNA binding activities. KEGG pathway bubble chart (Figure 5D, YlOrRd) identified enrichment in ribosome, coronavirus disease, and protein processing in endoplasmic reticulum pathways, with bubble size proportional to significance and numeric gene counts annotated. Key gene z‐score heatmap (Figure 5E, RdBu_r) revealed ISG15 as the most broadly expressed gene, with the highest z‐scores in stem‐like/mast cells (*z* = 1.50) and neutrophils (*z* = 0.60). TNFRSF18 showed strongest expression in stem‐like/mast cells (*z* = 1.93), whereas TNFRSF4 was specifically enriched in mast cells (*z* = 2.22). Key gene dot plot (Figure 5F, Viridis) confirmed these expression patterns across all seven immune cell types, with dot size reflecting detection rate and color intensity reflecting mean expression.

**Figure 5:**
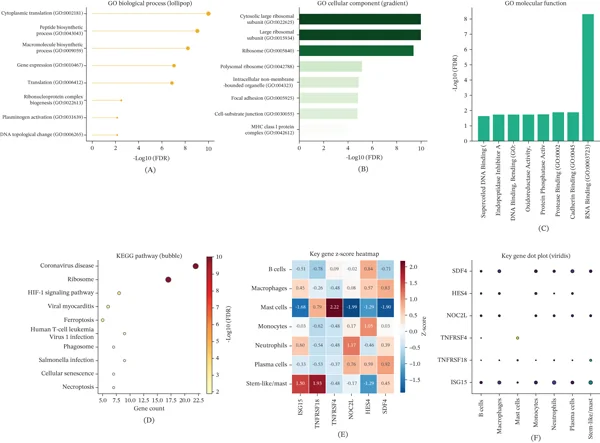
Functional enrichment and key gene expression analysis. (A) Lollipop chart of GO biological process enrichment (182 DEGs, Enrichr GO_2023). The Top 8 significantly enriched terms are shown; lollipop size scales with −log10(FDR). (B) Gradient horizontal bar chart of GO cellular component enrichment, with color intensity reflecting significance level (Greens). (C) Vertical bar chart of GO molecular function enrichment. (D) KEGG pathway enrichment bubble chart. Bubble size reflects −log10(FDR); color intensity (YlOrRd) reflects enrichment significance; numeric labels indicate gene count per pathway. (E) Z‐score normalized heatmap (RdBu_r) of six key genes (ISG15, TNFRSF18, TNFRSF4, NOC2L, HES4, and SDF4) across seven immune cell types. (F) Dot plot (Viridis colormap) showing average expression (color) and percent expressing cells (size) for the six key genes across cell types.

### 3.6. Key Gene Expression Profiling

Six key immunoregulatory genes were profiled using six distinct visualization types (Figure 6). NOC2L UMAP feature plot (Figure 6A, YlGn colormap) showed concentrated expression in B‐cell clusters, consistent with its transcriptional regulatory function in lymphocytes. HES4 violin plot (Figure 6B) exhibited scattered expression across multiple immune clusters, consistent with Notch signaling pathway activity in immune cell differentiation. ISG15 horizontal bar chart (Figure 6C) quantified mean expression across cell types, confirming its status as the most broadly and highly expressed immunoregulatory gene. TNFRSF18/GITR UMAP feature plot (Figure 6D, OrRd colormap) localized the sparse GITR‐positive cells within the UMAP embedding, without establishing a Treg‐specific function in this dataset. TNFRSF4/OX40 proportion bar chart (Figure 6E) quantified the percentage of OX40‐positive cells in each immune cell type, confirming highly restricted expression. SDF4 t‐SNE feature plot (Figure 6F, cool colormap) showed concentrated expression in B/plasma cell regions, consistent with its role in ER calcium binding and protein trafficking.

**Figure 6:**
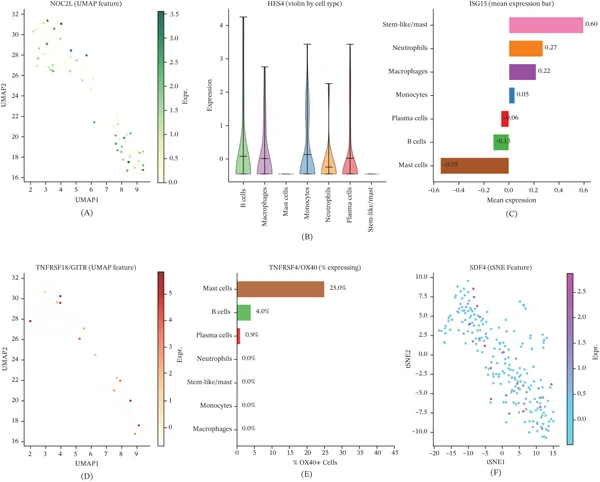
Key immunoregulatory gene expression profiles across immune cell types. Six distinct visualization types are used to characterize the expression patterns of six key immunoregulatory genes. (A) NOC2L: UMAP feature plot (YlGn colormap), showing concentrated expression in B‐cell clusters. (B) HES4: violin plot by cell type, showing scattered expression across multiple immune clusters, consistent with Notch signaling. (C) ISG15: horizontal bar chart of displayed ISG15 expression values by cell type; any negative values indicate scaled rather than log‐normalized expression and require confirmation against the figure source, confirming its status as the most broadly and highly expressed gene. (D) TNFRSF18/GITR: UMAP feature plot (OrRd colormap) highlighting the spatial localization of the few GITR‐positive cells. (E) TNFRSF4/OX40: horizontal bar chart showing percentage of OX40‐positive cells in each immune cell type, confirming highly restricted expression. (F) SDF4: t‐SNE feature plot (cool colormap), showing concentrated expression in B/plasma cell regions.

### 3.7. Pseudotime Trajectory and Key Gene Expression Dynamics

DPT analysis provided a descriptive ordering of heterogeneous immune cells (Figure 7). UMAP cell‐type reference (Figure 7A) provided spatial context for the pseudotime trajectory in the reduced dimensional space. Box plot analysis (Figure 7B) revealed pseudotime distribution differences across cell types, with macrophages extending to the maximum pseudotime value (1.0). Density ridge visualization (Figure 7C) showed that all seven cell types peaked at intermediate pseudotime values, with macrophages and plasma cells displaying the broadest temporal distributions. Smoothed trajectory analysis of ISG15, NOC2L, and HES4 (Figure 7D) revealed distinct temporal patterns: ISG15 maintained moderate expression throughout early‐to‐intermediate pseudotime with a slight decrease at later stages; NOC2L showed stable low‐level expression; HES4 exhibited enrichment at early pseudotime values. Scatter‐plus‐trend analysis of TNFRSF18, TNFRSF4, and SDF4 (Figure 7E) displayed individual cell‐level expression with LOESS/spline trend lines, suggesting visually similar intermediate pseudotime trends for TNFRSF18 and TNFRSF4 during intermediate pseudotime stages. Pseudotime‐binned heatmap (Figure 7F, coolwarm, 40 bins) illustrated similar smoothed patterns of TNFRSF18 and TNFRSF4, distinct from the broader expression profiles of ISG15 and SDF4, which do not establish statistical coregulation or developmental transitions.

**Figure 7:**
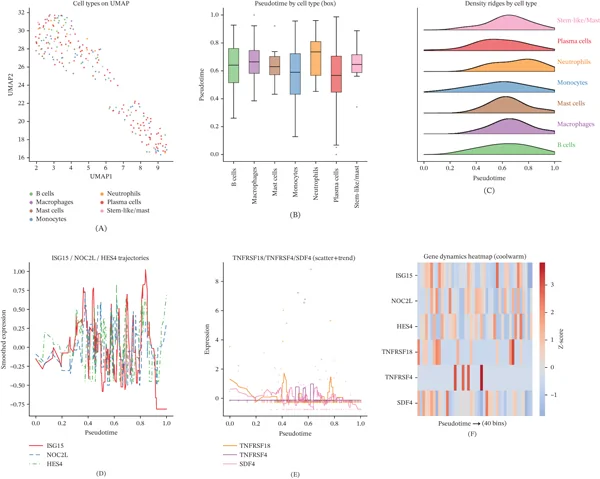
Descriptive pseudotime ordering and key gene expression patterns. (A) UMAP visualization colored by annotated cell type, serving as spatial reference for the pseudotime trajectory. (B) Box plot of pseudotime distribution by cell type, showing the range and quartiles of pseudotime values for each of the seven immune cell populations. (C) Density ridge plot of pseudotime distributions for each cell type. Filled curves are colored by cell type; curve height represents normalized density. (D) Smoothed expression trajectories (sliding window, window = 8) for ISG15 (red, solid), NOC2L (blue, dashed), and HES4 (green, dash‐dot) along pseudotime. (E) Scatter plot with LOESS/spline trend lines for TNFRSF18 (orange circles), TNFRSF4 (purple squares), and SDF4 (pink triangles) along pseudotime, showing individual cell‐level expression with overlaid smoothed trajectories. (F) Z‐score‐normalized heatmap (coolwarm) of six key gene expression profiles across pseudotime bins (the exact number of bins displayed requires verification against the source figure), showing descriptive expression trends of TNFRSF18 and TNFRSF4 at intermediate pseudotime stages.

## 4. Discussion

The scRNA‐seq analysis based on GEO dataset GSM4453577 provides preliminary descriptive observations into the immune microenvironment of lung cancer. Among the 11 identified immune cell subpopulations, ISG15 emerged as the most broadly and highly expressed key gene, showing prominent expression across all seven annotated immune cell subpopulations, with the highest z‐scores in stem‐like/mast cells (*z* = 1.50) and neutrophils (*z* = 0.60). ISG15 encodes a ubiquitin‐like modifier that is strongly induced by Type I interferons and plays critical roles in antiviral immunity, inflammation, and antitumor immune responses. The high expression of ISG15 across multiple immune cell types may be compatible with interferon‐related expression within the NSCLC TIME, potentially reflecting ongoing immune surveillance. High ISG15 expression has been associated with both favorable immune responses and resistance to cancer immunotherapy in various tumor types [8, 16].

TNFRSF18 (GITR) and TNFRSF4 (OX40) are costimulatory receptors belonging to the TNF receptor superfamily. Our analysis revealed that these two genes show visually similar trends during intermediate pseudotime stages, with TNFRSF18 showing the strongest expression in stem‐like/mast cells (*z* = 1.93) and TNFRSF4 specifically enriched in mast cells (*z* = 2.22), without demonstrating coregulation or a specific activation state. GITR signaling on regulatory T cells can modulate their suppressive function, and anti‐GITR therapy has emerged as a promising immunotherapeutic strategy in multiple cancers [9]. OX40 (TNFRSF4) costimulation enhances effector T‐cell survival and memory formation [10, 17]. The restricted expression pattern of TNFRSF4 in our analysis does not directly establish a cytotoxic T‐cell function because T‐cell markers were below detection.

HES4, a downstream target of Notch signaling, showed expression across multiple immune cell populations. Notch‐HES4 signaling is important for T‐cell lineage commitment and differentiation, and its expression in the TIME does not establish T‐cell differentiation in this T‐cell‐marker‐negative specimen [18]. NOC2L showed selective expression in B cells and macrophages. NOC2L functions as a transcriptional corepressor and has been implicated in ribosome biogenesis regulation. SDF4 (also known as CALNUC2) showed concentrated expression in B/plasma cell regions and may play roles in calcium signaling and protein trafficking in immune cells.

Functional enrichment analysis identified ribosomal biogenesis, protein processing in the endoplasmic reticulum, and cytoplasmic translation as the most significantly enriched biological processes. KEGG pathway analysis prominently identified terms including “coronavirus disease” and infectious disease–related pathways; these should not be interpreted as direct disease associations but rather as reflecting shared innate immune gene programs—particularly interferon signaling, ribosomal machinery, and antigen presentation pathways—that are broadly active across inflammatory contexts. Immune‐related pathway terms should be treated as exploratory observations, without prioritizing pathways unsupported by the reported leading enrichment results.

Cell–cell communication analysis suggested relatively high macrophage‐associated LR expression scores in the NSCLC tumor microenvironment, consistent with the known role of TAMs in shaping antitumor immunity [2]. The SPP1 signaling pathway analysis identified macrophages and monocytes as dominant signal senders, whereas neither MIF–CD74 nor SPP1 signaling activity was directly measured [7, 19]. MIF promotes tumor progression through multiple mechanisms, including angiogenesis promotion and immune suppression, whereas its receptor CD74 represents a potential therapeutic target [20].

The pseudotime trajectory analysis revealed that macrophages extend to the maximum pseudotime value (1.0) and, together with plasma cells, display the broadest temporal distributions, without establishing relative developmental maturity across distinct lineages. The provisional “stem‐like/mast” annotation is not sufficient to infer progenitor identity or an independent differentiation trajectory. The temporal regulation of TNFRSF18 and TNFRSF4 expression along pseudotime in specific immune states requires independent confirmation before therapeutic interpretation.

Key genes identified by scRNA‐seq analysis, including TNFRSF18 (GITR) and TNFRSF4 (OX40), represent candidates of potential therapeutic interest requiring functional validation before consideration as established immunotherapeutic targets.

This study has several important limitations. It examined one publicly available lung cancer specimen (223 retained cells) without biological replication, and rare annotated populations included only eight cells each. Thus, the observed cell‐type proportions and ISG15, TNFRSF18, and TNFRSF4 expression patterns cannot be generalized to NSCLC patients. Cell annotations were marker‐based and not independently validated, particularly for the provisional “stem‐like/mast” group. Dedicated doublet and ambient RNA analyses were not documented, and clustering stability was not tested. LR scores were expression‐based rather than validated signaling probabilities; pseudotime across unrelated immune lineages is descriptive and root‐dependent. The associations do not demonstrate lymph node metastasis, Treg activity, or immunotherapeutic efficacy. Independent multipatient data, orthogonal validation, and functional studies are necessary.

Future research should validate the reported immune cell annotations and candidate genes in independent NSCLC cohorts, test the robustness of clustering and lineage‐specific pseudotime analysis, and evaluate predicted LR interactions with established frameworks and orthogonal experiments.

## Author Contributions

Jianghua Mao and Xue Zhang contributed equally to this work; Rongwei Yang and Xiaolin Wang also contributed equally to this work.

## Funding

No funding was received for this manuscript.

## Conflicts of Interest

The authors declare no conflicts of interest.

## Data Availability

The data that support the findings of this study are available from the corresponding author upon reasonable request.
